# Commentary: Would your health be better if you lived in England or in the USA?

**DOI:** 10.1093/ije/dyu259

**Published:** 2015-02-19

**Authors:** David Stuckler, Aaron Reeves

**Affiliations:** ^1^Department of Sociology, Christ Church, Oxford University, Oxford, UK and ^2^Department of Sociology, Nuffield College, Oxford University, Oxford, UK

Would your health be better if you lived in England or in the USA? The study by Cieza (Cieza, unpublished for publication) and colleagues takes on this classic question, revisiting a powerful analysis from Banks and colleagues in 2006 that concludes the answer would have been (liberally interpreted): England is healthier.^1^

By way of background, the Banks *et al.* study compared rates of chronic diseases in middle-aged persons using two harmonized surveys, the English Longitudinal Study on Ageing (ELSA) and Health Survey for England, with the US Health and Retirement Survey (HRS) and National Health and Nutrition Examination Survey. People living in England had demonstrably better health profiles, irrespective of whether the chronic diseases were self-reported or diagnosed. This pattern also held for a series of biomarkers including plasma fibrinogen, glycosylated haemoglobin A1c (HbA1c), C-reactive protein and high-density lipoprotein cholesterol (HDL-C). Importantly, across the entire socioeconomic spectrum, the health of the English was better than that of North Americans. These data were more recently corroborated by the Global Burden of Disease 2010 study estimates, indicating that the life expectancy at age 50 for the English is 31.8 (healthy life expectancy, 25.2) and for North Americans is 31.1 years (healthy life expectancy, 24.7).^2^

The current study by Cieza *et al.* questions these data using the 2008 wave of ELSA and HRS, arguing that, by using chronic conditions, Banks and colleagues have missed out on the wider World Health Organization (WHO) definition of health—‘a state of complete physical, mental and social well-being and not merely the absence of disease or infirmity’—which some have argued can only be attained through the use of drugs. To better operationalize this wider definition, the study uses a psychometric approach, Rasch modelling, to create an integrated ‘health functioning scale’ ranging from 0 (worst) to 100 (best) health. This is based on survey respondents’ reports to 34 questions about perceived physical and cognitive functioning, and an additional six questions from measured tests of abilities. It also includes a wider sample, spanning ages 50–80.

In the first step, the study reproduces the original findings, showing that people living in England have a strong and significant health advantage. Using the 2008 data, Cieza and colleagues find that for diabetes, hypertension, heart disease, stroke, cancer and obesity, people living in England have lower rates than those living in the USA, with the exception of lung disease (likely reflecting a differential trajectory of smoking epidemics). It further verifies that this disadvantage is across the socioeconomic gradient; both high- and low-income North Americans have worse disease profiles. As one example, diabetes is prevalent in 20.2% of low-income Americans and 11.7% of high-income ones, whereas the corresponding English rates are 10.6% and 7.6%, respectively, considerably lower.

Next, to test their hypothesis that the English are not in fact healthier, having constructed the health functioning scale, the authors merge the USA and England datasets. They then regress the health scale on a dummy variable for whether the respondent was included the US survey, to test whether the English have higher scores on the scale. The unadjusted models are not shown, but their Table 4 models are displayed using corrections for the associations of a non-linear measure of age, sex and socio-economic measures (constrained to be similar in both nations—a debatable assumption, especially given differences in the education systems). Based on this analysis, the study reports that, ‘The English have a slightly better health than the Americans’ (p. 11).

Putting the magnitude of their estimated coefficient into perspective, they estimate that North Americans are 0.3% less healthy. This quantity broadly corresponds to those differences identified in GBD study, reported in Table 1.

**Table 1.** Comparison of life expectancy (LE) and healthy life expectancy (HALE), USA and UK, 2010

Age group	UK		USA		UK health advantage (difference)	
	LE	HALE	LE	HALE	LE	HALE
50-54	31.8	25.2	31.1	24.7	0.8	0.4
55-59	27.3	21.3	26.8	21.0	0.6	0.3
60-64	23.0	17.7	22.6	17.5	0.4	0.2
65-69	18.9	14.3	18.7	14.2	0.2	0.1
70-74	15.1	11.2	15.1	11.2	0.0	0.0

Source: GBD 2010.^2^

Before turning to the paper’s conclusions, several major limitations are worth noting. One is the authors’ reliance on self-reported measures. How do we know that the English and North American respondents will respond in culturally comparable ways? According to their analysis, the English compare favourably in more objective measures (such as hearing, seeing and mobility), whereas the Americans do better on those questions which are more subjective (such as self-reported energy levels, dizziness, pain and depression). In view of the observation that US and English respondents have similar self-reported health scores, perhaps this measure rather demonstrates what we have long known: that culture influences self-reported health.

Further, several components of the scale, such as for hearing ability, were generated with alternative thresholds for men and women (and even for each country) so that, even with similar answers to the survey questions, men and women would have differing health scores. Other measures, such as perceived energy levels, were taken from the HRS using a different number of threshold criteria from ELSA, so that the number of thresholds crossed (which is how Rasch models generate health scores) of the data will artefactually differ in England and the USA. The net effects of these multiple transformations will be to dilute differences between these countries—making such differences harder to detect should they actually exist.

Several curious reporting conventions are adopted. Why do the authors use 90% confidence intervals rather than the conventional 95%? In others, statistical statements are simply false. For example, the study compares the association of socioeconomic factors with their health scale, finding: ‘In both cases the variance explained was 17.7%, which confirms the tendency of no difference between both countries’. However, such goodness-of-fit tests do not permit such conclusions. The study decries the use of limited health dimensions in the original study, yet compiles information into its own unidimensional scale. It also constructs a straw man in its portrayal of previous cross-national work, failing to cite numerous well-conducted cross-national comparisons and falsely suggesting that the US National Research Council and Institute of Medicine Report based their conclusions on the Banks 2006 study.

Generally it is much harder to prove a null finding than a positive or negative one. The authors’ strong conclusion that the ‘English are at the end not healthier than Americans’ directly contradicts their own findings and statements. As documented here, the authors reproduce and confirm the Banks study: there exists an English health advantage that operates across the socioeconomic spectrum and this advantage cannot be explained by age, gender, income and education alone.

What are alternative explanations for the English health advantage? One, that has been reported elsewhere for avoidable mortality, is that North Americans do suffer a health disadvantage up to age 65, when they are eligible for Medicare, at which point their health begins to converge with that of people living in the UK. This could be tested using the authors’ data, by comparing ageing trajectories across these nations.

So should you live in the US or England? Judging on the health data alone, we find the weight of evidence still (slightly) favour England-but you might become more pessimistic about how healthy you feel.

## Funding

David Stuckler and Aaron Reeves are supported by a Wellcome Trust Investigator Award.
